# Knowledge, attitudes, beliefs and experience of palliative care amongst South African physiotherapists

**DOI:** 10.4102/sajp.v73i1.384

**Published:** 2017-07-31

**Authors:** Brenda M. Morrow, Charlotte Barnard, Zimkhitha Luhlaza, Kelisha Naidoo, Sarah Pitt

**Affiliations:** 1 Paediatrics and Child Health, University of Cape Town, South Africa; 2 Department of Health and Rehabilitation Sciences, University of Cape Town, South Africa

## Abstract

**Background:**

Palliative care encompasses holistic management of patients and families facing life-threatening and life-limiting conditions. There is currently little known about South African physiotherapists’ palliative care knowledge, attitudes, beliefs, experience and training needs.

**Objectives:**

To describe the amount and adequacy of palliative care training received by South African physiotherapists, and their interest, knowledge, attitudes, beliefs and experiences of palliative care.

**Methods:**

This was a cross-sectional descriptive survey study of a convenience sample of physiotherapists, using an adapted Physical Therapy in Palliative Care-Knowledge, Attitudes, Beliefs and Experiences Scale (PTiPC-KABE Scale). Likert scale scores were converted to continuous data for analysis, presented as median (IQR). Seven universities were contacted to determine undergraduate palliative care curriculum content.

**Results:**

A total of 303 participants (8.4% response rate) completed the questionnaire, and 289 responses were included (5.35% margin of error with 95% CI). Participants had 16 (6–27) years of experience, with 85.5% in private practice. About 66.7% and 79% of participants reported not receiving any training at undergraduate and postgraduate levels, respectively, with more than 80% expressing that training was inadequate at both levels. Universities (*n* = 4/7; 57.1%) reported a maximum 3 hours undergraduate palliative care training. Seventy-nine percent of respondents had clinical experience in providing palliative care; however ‘knowledge’ was the lowest scoring domain (56.3% (43.8%–62.5%). The ‘beliefs’ domain scored highest at 82.6% (69.6%–91.3%).

**Conclusion:**

Many South African physiotherapists manage patients requiring palliative care, despite inadequate training and limited knowledge in this field. More under- and postgraduate learning opportunities should be made available for physiotherapists in the area of palliative care.

## Introduction

Palliative care is a holistic practice discipline involving the care of people and their families faced with life-threatening and life-limiting illnesses (McMillan et al. 2014). Palliative care aims to improve quality of life through prevention, early identification and relief of physical (including pain), psychological, psychosocial and spiritual suffering and by optimising independent function (Barnard & Gwyther 2006; World Health Organization 2015).

The palliative care approach affirms life, while regarding dying as a normal process (World Health Organization 2015). It aims neither to hasten nor postpone death and can be used in conjunction with curative therapies aimed at prolonging life (e.g. chemotherapy and antibiotics) (World Health Organization 2015). Palliative care, therefore, encompasses care from the point of diagnosis (Murray et al. 2015) and includes, but is not limited to, end of life care, with changing aims according to disease progression.

Advances in medical care have resulted in a greater number of people reaching old age and living with health conditions common in the elderly (Payne, Coyne & Smith 2002). Between 1996 and 2011, the number of people aged 60 years and above rose in South Africa from 2.8 to 4.1 million individuals (Statistics South Africa 2014). Furthermore, a number of chronic, life-threatening conditions are prevalent in South Africa, including HIV, cerebrovascular disease, diabetes mellitus and chronic pulmonary disease (Statistics South Africa 2014). People presenting with these conditions or their sequelae could benefit from a palliative care approach.

Considering the diverse needs of individual patients and their families, a multidisciplinary team approach to palliative care is appropriate (Veqar 2013), with an emphasis on effective communication amongst all team members, including the patient and family (Buckman 2001). Physiotherapy has an important role in the palliative care team, providing symptom management and improving quality of life by optimising independent levels of function (Alice & Mulle 2011; Barawid et al. 2015; Frymark, Hallgren & Reisberg 2010; Kumar & Jim 2010). Specific physiotherapy modalities used in palliative care aim to: reduce pain; reduce fatigue, dyspnoea and other respiratory symptoms; decrease spasticity and other neuromuscular problems; strengthen or maintain muscle strength; prevent postural deformities which may impact on function; reduce functional limitations and improve self-sufficiency, self-motivation, social interaction and respiratory function (Kumar & Jim 2010). Palliative care rehabilitation has been positively associated with a high and prolonged level of independent function (Cobbe et al. 2013), which also reduces the burden on care providers (Downing et al. 2011).

As professionals in this field, physiotherapists should have sufficient knowledge, experience and skills to manage patients with difficult diagnoses at different life stages (Chiarelli, Johnston & Osmotherly 2014). However, there is currently little known about the experience, attitudes, beliefs and knowledge of palliative care amongst South African physiotherapists, and existing studies mainly relate to undergraduate physiotherapy students (Kumar, Jim & Sisodia 2011; Morris & Leonard 2007).

Kumar et al. (2011) reported that introducing a new palliative care training programme brought about a significant positive change in palliative care knowledge, attitudes, beliefs and experiences amongst student physiotherapists in a single centre in India (Kumar et al. 2011). Outcomes were measured using a questionnaire, the Physical Therapy in Palliative Care-Knowledge, Attitudes, Beliefs and Experiences Scale (PTiPC-KABE scale). Although this questionnaire was found to be reliable, based on a test-retest pilot, validity could not be guaranteed, and several ambiguities (possibly relating to language translation issues) in the questionnaire may have impacted on the results. Considering the cultural and sociodemographic differences between India and South Africa, the results of this study cannot be directly extrapolated to the South African context.

Another small, single centre, qualitative study (*n* = 6) described positive learning experiences of final year student physical therapists within a clinical multidisciplinary palliative care team placement (Morris & Leonard 2007). Authors reported that the placement helped students to identify their role in palliative care, to see the effects of their contribution, and to improve communication and build relationships with patients, which are essential components of effective and successful rehabilitation. The extent and type of undergraduate physiotherapy training in palliative care amongst different tertiary learning institutions in South Africa is currently not known.

## Aims and objectives

This study aimed to investigate the existing knowledge, attitudes, beliefs, training and experiences of palliative care by physiotherapists working in South Africa.

Specific objectives were:

- To determine what proportion of South African physiotherapists
  ■ had received under- and postgraduate training in palliative care, and perceptions of training adequacy
  ■ had clinical experience of palliative care
- To determine overall interest in palliative care amongst South African physiotherapists
- To describe prevailing knowledge, beliefs, attitudes and experiences of palliative care amongst South African physiotherapists
- To describe current undergraduate training courses in physiotherapy palliative care, as provided by South African university physiotherapy departments.

## Methodology

### Research design

This was a quantitative cross-sectional descriptive study using an online questionnaire.

### Participants

A convenience sample of anonymised participants was recruited from the South African Society of Physiotherapy (SASP) membership database. Male and female participants were eligible for inclusion if they had obtained a degree in physiotherapy in South Africa (including 2015 graduates) and were currently residing and practising physiotherapy in South Africa. Physiotherapists who were not members of the SASP and otherwise eligible participants who did not have access to email were excluded from participation.

A sample size of 347 participants was calculated as being required in order to obtain statistically significant results, with a confidence interval of 95% and a margin of error of 5%, based on a population (SASP membership) of approximately 3500 and a 20% predicted response rate.

### Measurement instrument

A minimally adapted version of the PTiPC-KABE scale was used. This scale has previously been determined to be reliable, and is in the process of being validated (Kumar et al. 2011). The original PTiPC-KABE scale contained both quantitative and qualitative questions. The locally adapted version for this study mainly contained quantitative questions and additionally provided opportunity for respondents to elaborate on their answers in free-text boxes. The questionnaire was predominantly unchanged overall, but questions regarding interest and training received in palliative care were added. Some of the questions from the PTiPC-KABE scale questionnaire used by Kumar et al. (2011) were reworded to be less ambiguous thereby reducing risk of bias.

The PTiPC-KABE scale questions were categorized into four sections according to whether they evaluated knowledge, attitudes, beliefs or experiences. The total possible score achieved was 85, with total possible individual domain scores of 16 (knowledge), 6 (attitudes), 23 (beliefs) and 42 (experience). Scores are presented as a percentage of total throughout. Survey Monkey (https://www.surveymonkey.com/) was used to build the online survey.

### Procedure

After initial adaption, the questionnaire was sent to two experts in the field of palliative care to gain content validity. Further minor changes were made from this feedback before an invitation email with information sheet and link to the online questionnaire was distributed to the SASP membership by the SASP secretariat. Two reminder emails were sent prior to the due date.

An email was also sent to the departments of seven universities which offer a physiotherapy degree in South Africa, with a request for them to send details of the number of hours and curriculum guidelines regarding palliative care training at undergraduate and postgraduate levels.

### Analysis

Responses were scored as described by Kumar et al. (2011), using the Likert scoring system (strongly agree, somewhat agree, neutral, somewhat disagree, disagree, N/A). The scores, ‘strongly agree’ and ‘somewhat agree’ were combined into ‘agree’, and ‘strongly disagree’ and ‘somewhat disagree’ into ‘disagree’. Points were awarded as follows: 3 points to ‘agree’, 2 to ‘neutral’, 1 to ‘disagree’ and 0 to N/A or no response. The true or false questions were awarded scores of 1 or 0. This was done to develop continuous data for computational purposes. The scores were inverted for items with a likely negative impact on provision of palliative care, as described previously by Kumar et al. (2011).

Data were tested for normality using the Shapiro Wilks W test. Descriptive continuous variables are presented as median (interquartile range, IQR) as appropriate for nonparametric data. Categorical data are presented as *n* (%). Associations between the categories of years of experience, training received (under- and postgraduate) and areas of practice, with the overall and individual domain scores of the PTiPC-KABE scale, were assessed using Kruskall-Wallis ANOVA. Spearman *R* correlation tests were used to evaluate associations between the number of years’ experience (continuous variable) and domain scores. A significance level of less than 0.05 was chosen, and Statistica (version 12, Statsoft Inc., USA) was used for all analyses.

### Ethical consideration

On entering the survey website, participants were provided with information regarding the purpose of the study, risks and benefits, their right to withdraw from the study at any time without reason and with no consequence, and that confidentiality and anonymity would be ensured. No identifiers were obtained or recorded as part of this study. Although the informed consent form was not physically signed, participants were informed that clicking the link and accessing the questionnaire was taken as providing consent.

The study was approved by the institutional Health Research Ethics Committee (HREC Rec/Ref 181/2016) and adheres to the principles outlined in the Declaration of Helsinki 2013.

## Results

The survey link was sent to 3609 post-qualification SASP members, of whom 303 (8.4%) completed the survey online. Fourteen (4.6%) participants were subsequently excluded as they had qualified outside South Africa, leaving 289 participants for analysis. This sample size gives a margin of error of 5.35%, with a 95% confidence interval.

**FIGURE 1:**
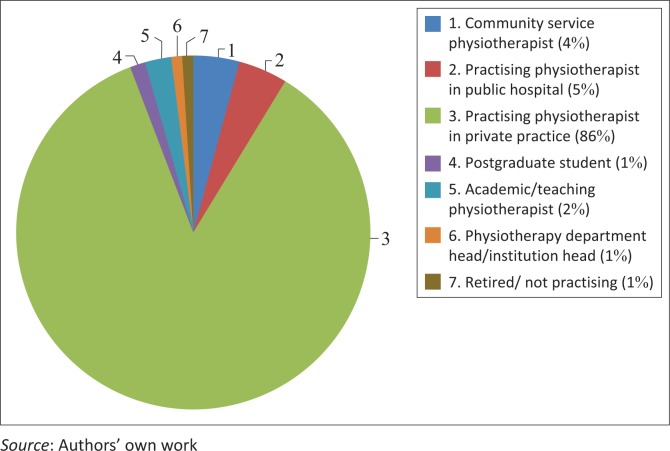
Professional positions held by respondents (*n* = 289).

Participants had median (interquartile range, IQR) 16 (6–27) years of experience as a physiotherapist, with the majority (*n* = 67; 23.2%) having qualified between 2011 and 2015 (Table 1). Most respondents (*n* = 247; 85.5%) practised physiotherapy in a private practice setting (Figure 1).

**TABLE 1:** Year qualified as a physiotherapist (*n* = 289).

Qualification year	*n* (%)
2011–2015	67 (23.2)
2006–2010	41 (14.2)
2001–2005	36 (12.5)
1996–2000	37 (12.8)
1991–1995	22 (7.6)
1986–1990	37 (12.8)
1981–1985	20 (6.9)
1976–1980	15 (5.2)
1971–1975	9 (3.1)
1970 or earlier	5 (1.7)

*Source:* Authors’ own work

**FIGURE 2:**
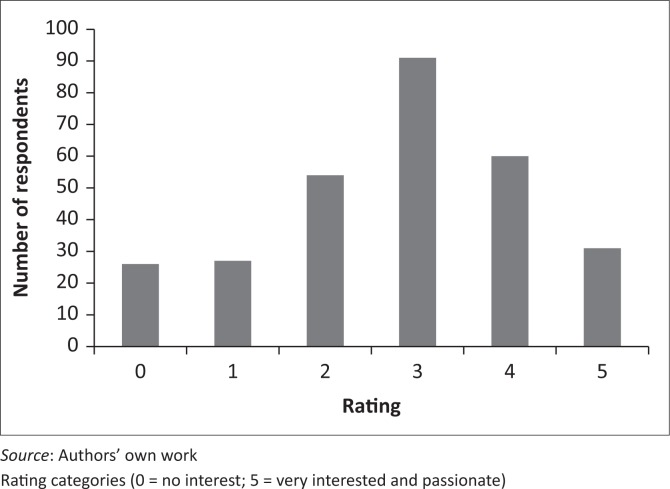
Overall interest in palliative care in physiotherapy, rated on a five-point Likert scale (*n* = 289).

On a five-point Likert scale, the average rating of overall interest in palliative care in physiotherapy was 3.8 with most participants (*n* = 91, 31.5%) being ‘somewhat interested’ in the field, with a score of 3 (Figure 2).

The open-ended question related to defining palliative care suggested that the majority of respondents understood that palliative care referred to holistic care (including emotional, psychological, medical and physical care of patients and their families) aimed at improving comfort, pain relief and quality of life of seriously ill patients. However, many respondents (*n* = 54, 18.7%) defined palliative care more narrowly as being limited to end-of life care.

Of the 279 (96.5%) responses to the question related to undergraduate training, 184 (66.7%) reported that they had not received any training at this level and 231 (83.7%) felt that undergraduate training in palliative care in physiotherapy was inadequate.

Four of the seven (57.1%) university physiotherapy departments approached responded to the email sent regarding undergraduate course content (Table 2). Of the responding institutions, the maximum time dedicated to training in palliative care in physiotherapy was 3 hours.

**TABLE 2:** Responses from universities (*n* = 7) with regard to specified palliative care training and instruction.

University code	Number of hours of specified palliative care training in physiotherapy	Comments
A	1	Lectures are given in second year as part of the Clinical Science course.
B	3	In third year students receive a 2 h lecture on how to handle death of a patient and a 1-h lecture on the management and palliative care of a lung cancer patient.
C	0	Palliative care education is covered when studying specific conditions. This institution is in the process of revising their curriculum after the academic staff identified a gap and are outsourcing a topic to be taught by a physiotherapist specifically trained in oncology and palliative care.
D	Did not respond.	-
E	Did not respond.	-
F	3	Fourth year students have two 1.5-h class based discussion entitled ‘The Meaningful Life and Death’.
G	Did not respond.	-

*Source:* Authors’ own work

In the related open-ended question, respondents highlighted the following main themes, which should be given priority at an undergraduate training level:

- definition and scope of palliative care (*n* = 13),
- the psychology and emotional response to palliative care, end of life processes and bereavement (for the therapist, patient and family) (*n* = 16),
- communication, including counselling skills and truth-telling (*n* = 9),
- ethical considerations (*n* = 7),
- role of the physiotherapist in the palliative care team, including indications, contraindications and specific techniques (*n* = 13).

At a postgraduate level, 56 of 267 (21.0%) respondents had received specific postgraduate training in palliative care. The related open-ended questions suggested that the majority of postgraduate training received was in the form of continuous professional development (CPD) lectures and as part of short courses. The majority of respondents (*n* = 219, 82.0%) felt that postgraduate training in palliative care in physiotherapy was inadequate.

**FIGURE 3:**
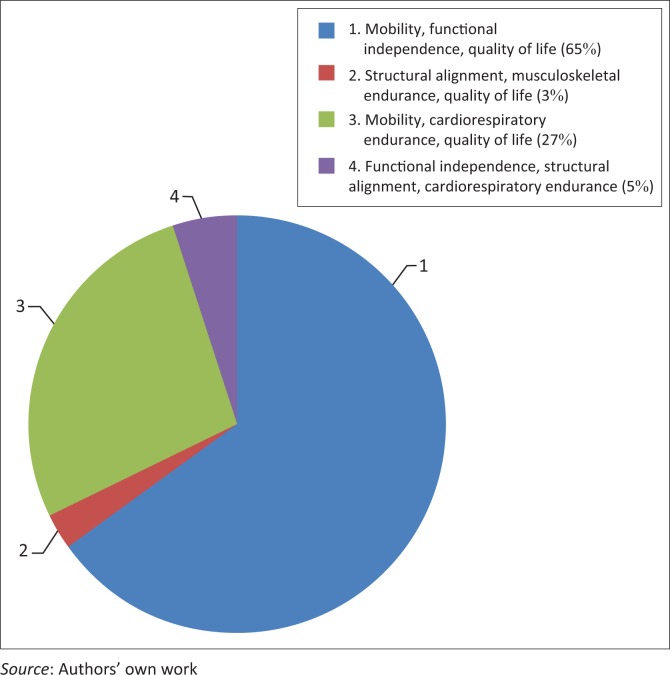
Perception of the most important focus or goal of physiotherapy management in palliative care (*n* = 184).

Of 184 (64%) respondents, 119 (65%) perceived improved mobility, functional independence and quality of life as the most important aspect or aim of physiotherapy management in palliative care (Figure 3).

**FIGURE 4:**
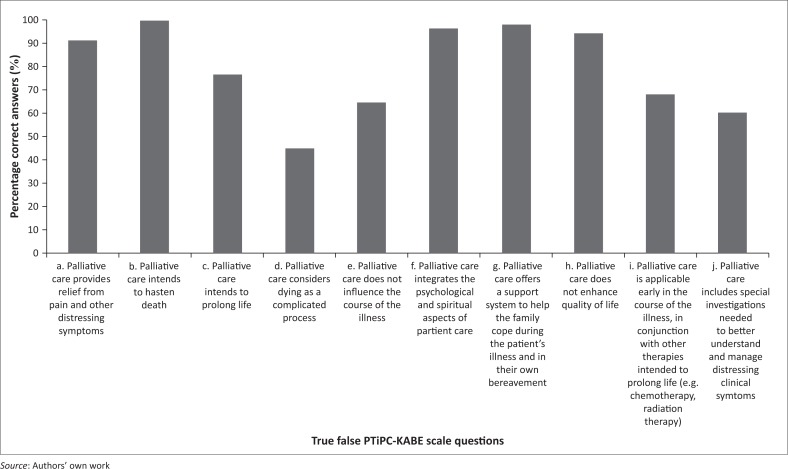
Percentage correct responses to the ‘true or false’ questions of the PTiPC-KABE scale (Question 11).

Two hundred and thirty-four (81.0%) respondents completed the PTiPC-KABE scale questions related to experiences, knowledge, attitudes and beliefs, with the highest scores attained in the ‘belief’ domain (Table 3). Specific answers to Likert scale questions and true–false questions are presented in Table 4 and Figure 4, respectively.

**TABLE 3:** Perceptions and experiences of palliative care in physiotherapy, using the PTiPC-KABE scale (*n* = 234).

Domain	Median (IQR) score achieved (% total ideal score)
Total score	67.1 (58.8–76.5)
Knowledge	56.3 (43.8–62.5)
Attitudes	66.7 (50.0–83.3)
Beliefs	82.6 (69.6–91.3)
Experience	64.3 (50.0–76.2)

*Source:* Authors’ own work

IQR, interquartile range.

No significant associations were found between training received and areas of practice and either overall or individual domain scores of the PTiPC-KABE scale. The only domain significantly associated with years of experience was that of ‘attitudes’, with a negative correlation between number of years qualified and percentage score (Spearman *R* = −0.14; *p* = 0.03).

## Discussion

To our knowledge, this is the first study to investigate the training levels, attitudes, beliefs, experiences and knowledge of palliative care amongst practising physiotherapists in South Africa. Overall responses indicate that although most physiotherapists who responded had clinical experience in palliative care, training in this area was inadequate, with variable responses in terms of individual domains.

The majority of participants reported being ‘somewhat interested’ in palliative care in physiotherapy, with a small proportion (*n* = 31; 10.7%) being very interested and a similar proportion (*n* = 26; 9.0%) having no interest in the field. Despite the overall moderate interest in the field of palliative care, almost 80% of respondents had clinical experience in palliative care (Table 4), and the vast majority felt that palliative care training received at both under- and postgraduate levels was inadequate. This perception is supported by the university respondents, who only allocated a maximum of 3 hours undergraduate teaching to this field, with no standardisation amongst different institutions. One university department had identified this area as a gap in the undergraduate curriculum and was in the process of curriculum revision to include palliative care. No conclusions can be made regarding the palliative care teaching provided at those universities that did not respond to the mail query. Participants suggested topics that should be prioritised at undergraduate level, including psychological and emotional responses to palliative care, end of life processes and bereavement; definitions and scope of palliative care; and the specific role of the physiotherapist in palliative care. These suggestions could be considered in curriculum development processes. In addition, although approximately two-thirds of participants were able to correctly identify some of the most important physiotherapy aims of treatment in palliative care (improving mobility, functional independence and quality of life) (Chiarelli et al. 2014), there was a wide range of individual responses. Educating physiotherapists on appropriate clinical goal setting in palliative care practice is also a potentially important component of the undergraduate curriculum.

**TABLE 4:** Summarised responses to questions (*n* = 234) related to experience and beliefs of physiotherapy in palliative care, using the PTiPC-KABE scale (Question 7).

Responses	Neutral *n* (%)	Agree *n* (%)	N/A *n* (%)	Disagree *n* (%)
a. Palliative care is as important as curative care in physiotherapy practice.	39 (16.7)	176 (75.2)	0 (0.0)	19 (8.1)
b. I have had experience providing palliative care to patients and their families.	18 (7.7)	186 (79.5)	10 (4.3)	20 (8.5)
c. I feel a sense of personal failure when a patient dies.	39 (16.7)	65 (27.8)	6 (2.6)	124 (53.0)
d. There is societal support for and awareness of the need for physiotherapy in palliative care.	33 (14.1)	58 (24.8)	3 (1.3)	140 (59.8)
e. The medical staff supports physiotherapy in palliative care in my workplace.	48 (20.5)	91 (38.9)	40 (17.1)	55 (23.5)
f. The physical environment of my workplace is ideal for providing palliative care and rehabilitation.	38 (16.2)	93 (39.7)	28 (12.0)	75 (32.1)
g. My workplace is adequately staffed for providing palliative care (including palliative rehabilitation) to patients and their families.	39 (16.7)	74 (31.6)	27 (11.5)	94 (40.2)
h. In my workplace, families are involved in decision making about the terminally ill patient.	30 (12.8)	135 (57.7)	36 (15.4)	33 (14.1)
i. Providing pain relief is a priority to me, when patients are nearing the end of life.	13 (5.6)	194 (82.9)	23 (9.8)	4 (1.7)
j. I am often exposed to death in my workplace.	24 (10.3)	130 (55.6)	28 (12.0)	52 (22.2)
k. Palliative care is necessary in physiotherapy education.	10 (4.3)	201 (85.9)	20 (8.55)	3 (1.3)
l. When a patient under my care dies, I have sufficient time to spend with his or her family.	37 (15.8)	60 (25.6)	33 (14.1)	104 (44.4)
m. There are policies and guidelines to assist delivery of palliative care in my workplace.	39 (16.7)	37 (15.8)	35 (15.0)	123 (52.6)
n. In my workplace, when a diagnosis with a poor outcome is made, the patient and his or her family are informed of palliative care options.	39 (16.7)	109 (46.6)	35 (15.0)	51 (21.8)
o. In my area of work, the multi-disciplinary team expresses and discusses its opinions, values and beliefs about palliative care.	37 (15.8)	70 (29.9)	46 (19.7)	81 (34.6)
p. Caring for dying patients is traumatic for me.	46 (19.7)	109 (46.6)	12 (5.1)	67 (28.6)
q. I have received education that assists me to support and communicate with terminally ill/ dying patients and their families.	30 (12.8)	61 (26.1)	14 (6.0)	129 (55.1)
r. All members of the healthcare team in my workplace agree with and support palliative care when it is implemented for the terminally ill patient.	56 (23.9)	95 (40.6)	50 (21.4)	33 (66.9)
s. In my workplace, staff go beyond what they feel comfortable with in using technological life support.	57 (24.4)	45 (19.2)	67 (28.6)	65 (27.8)
t. In my workplace, staff are asked by the family to continue life-extending care beyond what they feel is right.	50 (21.4)	59 (25.2)	65 (27.8)	60 (25.6)
u. My personal attitudes about death affect my willingness to deliver palliative care.	40 (17.1)	65 (27.8)	17 (7.3)	112 (47.9)
v. Palliative care is against the values of physiotherapy.	11 (4.7)	11 (4.7)	4 (1.7)	208 (88.9)
w. When a patient dies in my workplace, counselling is available for me if I need it.	26 (11.1)	46 (19.7)	37 (15.8)	125 (53.4)
x. There is a belief in my society that patients should not die, under any circumstances.	21 (9.0)	30 (12.8)	9 (3.8)	174 (74.4)
y. Curative care is more important than palliative care in the physiotherapy environment.	46 (19.7)	62 (26.5)	4 (1.7)	122 (52.1)

*Source:* Authors’ own work

Only 21% of respondents had received any postgraduate training in palliative care, mostly through short educational courses and lectures. Postgraduate course attendance requires purposive selection in an area of perceived need. Some participants stated that it was difficult to identify physiotherapy-specific palliative care courses. Although asked, none of the universities in correspondence mentioned any postgraduate palliative care in physiotherapy programmes. In other healthcare professions, undergraduate and postgraduate palliative care education has been shown to positively benefit individuals, with reduced fear of death (Mason & Ellershaw 2004) and improved self-efficacy in palliative care symptom management (Adriaansen, Van Achterberg & Borm 2005).

A number of web-based educational postgraduate courses and general palliative care educational programmes are available for South African physiotherapists, which could be highlighted by physiotherapy special interest groups. Examples of these include an online module on the promotion of the role of physiotherapy in palliative care, which is available for self-study or as part of a continued professional development course (McMillan et al. 2014). St Luke’s Hospice also offers general short courses in palliative care in South Africa (http://www.stlukes.co.za/health-care-training-st-lukes-hospice/). In addition, postgraduate programmes in palliative care are available at some universities, albeit not specifically targeting physiotherapy practice.

Although many respondents correctly described palliative care as being holistically oriented, a large proportion of respondents defined palliative care narrowly as being limited to ‘end of life’ care. This suggests that the knowledge of the scope of palliative care is still limited, and this suggestion is supported by the median ‘knowledge’ domain score of only 56%. Reasons for this low knowledge score may include the inadequate training currently provided at an undergraduate level and the lack of training opportunities at a postgraduate level, as described above. Previous studies have highlighted the importance of improved knowledge through education in healthcare practice, including physiotherapy. Improved knowledge allows better understanding of and engagement with presenting patients’ problems, improves communication and enhances clinical decision making skills in palliative care (Nairn 2012; Zimny & Tandy 1989). A nursing education study showed that individualised patient care is most effective when knowledge and experience are integrated (Skar 2010).

Regarding perceptions of palliative care in physiotherapy, the ‘beliefs’ domain scored the highest overall (median 82.6%). Results suggest that many participants rely on their beliefs (including self-developed morals and values) rather than knowledge to guide them in the management of patients requiring palliative care. Furthermore, the scores obtained suggest that prevailing beliefs by physiotherapists are supportive of the palliative care approach. The domains of attitudes and experiences (66.7% and 64.3%, respectively), which are essentially self-developed, also ranked higher than knowledge. Self-efficacy is the result of the combination of beliefs and acquired knowledge and skill (Jones & Sheppard 2011). The implication of this is that without adequate knowledge and taught skill of the physiotherapist’s specific role in palliative care, physiotherapists may not perform at an appropriate standard.

‘Attitude’ to palliative care was negatively associated with years of experience. The reasons for this are unclear, but may reflect the inability of physiotherapists to cope with the demands of palliative care patients over time. This may relate to the inadequacy of training in the field, which also may translate into inadequate support structures being in place for emotional support of all staff working in the area. It is concerning that 28% of respondents reported feeling a personal sense of failure when a patient died, and 53% of respondents indicated that counselling was not available for them in the event of a patient death. Without adequate support structures in place, physiotherapists may find this particular field of practice emotionally and physically exhausting, with the possible consequence of burnout, as has been observed in other healthcare fields (Gillman et al. 2015; Rushton, Kaszniak & Halifax 2013). This requires further investigation in the physiotherapy profession.

It is interesting that 60% and 24% of respondents, respectively, disagreed with the statements ‘There is societal support for and awareness of the need for physiotherapy in palliative care’ and ‘The medical staff support physiotherapy in palliative care in my workplace’. It is, therefore, recommended that awareness of the role of physiotherapy in palliative care should be highlighted amongst other members of the healthcare team, educators, advocacy groups and the community.

### Limitations

While the survey response rate and margin of error were reasonable, we did not reach the intended sample size. Non-response could relate to changes of contact information, members not regularly checking their emails or too many requests for completion of e-surveys.

Only members of the SASP were approached for inclusion in the study, and the majority of respondents were working in private practice, raising the possibility of selection bias. Therefore these results require confirmation in a larger sample with greater representivity. The survey runs the risk of recall bias; however, considering that the largest number of responses were from participants who graduated after 2011, recall bias related to undergraduate training received is unlikely to be a significant factor.

## Conclusion

Palliative care is an important component of physiotherapy practice, yet there is little available literature in the South African context. Based on the findings of this study, it can be understood that a large proportion of South African physiotherapists have clinical experience managing patients requiring palliative care, despite inadequate training and limited knowledge in this field. It is suggested that more postgraduate learning opportunities be made available, and that more attention be paid to this clinical area during undergraduate training. South African physiotherapists generally have attitudes and beliefs which support palliative care, with more recently qualified physiotherapists tending to have better attitudes in this regard. There is a need for increased awareness regarding the role of physiotherapists in palliative care in South Africa, improved support of physiotherapists practising in the field and more research on the role of physiotherapy in palliative care is warranted.
